# Identification of dengue type 2 virus in febrile travellers returning from Burkina Faso to France, related to an ongoing outbreak, October to November 2016

**DOI:** 10.2807/1560-7917.ES.2016.21.50.30425

**Published:** 2016-12-15

**Authors:** Carole Eldin, Philippe Gautret, Antoine Nougairede, Mélanie Sentis, Laetitia Ninove, Nadia Saidani, Matthieu Million, Philippe Brouqui, Remi Charrel, Philippe Parola

**Affiliations:** 1 URMITE, Aix Marseille Université (UM63, CNRS 7278, IRD 198, INSERM 1095, IHU - Méditerranée Infection), Marseille, France; 2 UMR ‘Emergence des Pathologies Virales’ (EPV: Aix-Marseille Univ - IRD 190 - Inserm 1207 - EHESP), Marseille, France

**Keywords:** Dengue fever, Africa, outbreak, Burkina Faso, traveller

## Abstract

Dengue fever is rarely reported in travellers returning from Africa. We report two cases of dengue fever in travellers returning from Burkina Faso to France. One of them presented a severe dengue fever with ALT > 1,000 IU/L and pericarditis. Serotype 2 was identified. The cases reflect a large ongoing outbreak with over 1,000 reported cases between August and November in the capital city. Clinicians should consider dengue fever in malaria-negative febrile travellers returning from Africa.

Dengue fever in returning travellers to non-endemic areas has been mainly reported after visits to South-east Asia, Central Asia, or South America [1]. In Africa, its epidemiology is poorly described even if the disease has long been known to exist [2]. We report two cases of dengue fever identified in travellers returning from Ouagadougou, Burkina Faso, to Marseille, France, in late autumn 2016, reflecting a large ongoing local outbreak.

## Case descriptions

The first case was a woman in her mid-twenties, who travelled to Burkina Faso as a logistician for a medical non-governmental organisation (NGO). She spent 10 days in Ouagadougou from 23 October to 3 November 2016 and used atovaquone/proguanil for malaria prophylaxis. Three days after her arrival, she developed fever, headache, myalgia, nausea and diarrhoea. Two days after she came back to France, she presented at our centre with persisting diarrhoea on day 9 after symptom onset. At examination she had no fever, complained of weakness and had a painful abdomen. Dengue nonstructural protein 1 (NS1) antigen (Ag) and serology (IgM) was negative (SD BIOLINE Dengue Duo Combo Device, Standard Diagnostics Inc, Korea) and malaria rapid diagnostic tests (Palutop+4, All. Diag, France) were negative, as was serology with an in-house MAC ELISA for IgM and in-house indirect ELISA for IgG [3]. A serum sample was positive for dengue viral RNA [4] and typed as dengue 2 viral RNA [5]. Blood and stool cultures, parasitological examination of stools and RealStar Chikungunya RT-PCR kit 1.1 (Altona Diagnostics, Germany) were negative. Convalescent serum was not collected because the patient did not attend the follow-up visit (Table).

**Table:** Laboratory test results, two cases of dengue fever in travellers returning from Ouagadougou, Burkina Faso, to Marseille, France, October to November 2016^a^

**Table:** 

Laboratory results (norm)	Case 1	Case 2
Leukocyte count/μl (4,000-10,000)	1,650	2,700
Platelet count/μl (150,000-400,000)	296,000	142,000
ALT/AST IU/L (10-40)	62/53	1,800/NA
Dengue NS1 Ag (NA)	Negative	Positive
Dengue serology (IgM and IgG)^b^ (NA)	Negative	Negative
Real-time RT-PCR detecting all dengue virus RNA (NA)	Positive	Negative
Type specific real-time RT-PCR (NA)	Positive serotype 2	Negative

Ag: antigen; ALT: alanine transaminase; AST: aspartate aminotransferase; NA: not available NS 1: non-structural protein RT: reverse transcription.

^a^ Only values deviating strongly from the norm are presented.

^b^ MAC ELISA for IgM and in-house indirect ELISA for IgG [3].

The second case was a woman in her 50s who travelled from 12 October to 10 November 2016 to Ouagadougou, where she worked with the organisation of a theatre festival. One week after arrival, she presented fever up to 40.5 °C, arthralgia and diarrhoea. She presented at a local medical centre and was treated by quinine for malaria without blood tests performed. Fever and diarrhoea persisted and on day 3 of illness dark urine appeared. She thus consulted the International Medical Center in Ouagadougou where blood sample analysis revealed severely elevated liver transaminases (Table). Malaria was ruled out by microscopic blood smear examination and Dengue NS1 Ag testing was performed and found positive. She came back to France and consulted a cardiologist because of chest pain. Chest computerised tomography (CT)-scan ruled out pulmonary embolism and pericarditis was diagnosed by echocardiography. She presented at our centre two weeks after resolution of symptoms because she had questions about the prevention of dengue fever.

**Epidemiological situation in Burkina Faso and neighbouring countries**

The first dengue fever outbreak in Burkina Faso was reported in 1925 [2]. In 1982, a second outbreak was described between September and December, with 30 cases reported (mainly European expatriate patients), and two strains of dengue virus serotype 2 were isolated for the first time in this country [6]. In 2013, another epidemic occurred between October and November, and serotype 3 was isolated [7]. On 18 November 2016, the World Health Organization (WHO) reported 1,061 suspected cases of dengue fever in Burkina Faso between August and November 2016, including 15 fatal cases [8]. Serotype 2 has been identified in the current outbreak, but further investigations are in progress. 

Concerning other West African countries, data are scarce. The last epidemic in Senegal was reported in 2009, caused by dengue type 3 virus [9]. In Sierra Leone, Mali and the Ivory Coast, seroprevalence studies among febrile patients reflect the circulation of the virus [10-12]. Cases from Togo and Benin have been reported only in travellers [2,13]. 

## Discussion

We report two cases of dengue fever in travellers returning from Burkina Faso to France in late autumn 2016. The first patient had a non-complicated dengue fever according to the WHO dengue fever classification criteria [14]. The second one fulfilled the criteria for severe dengue fever, with ALT > 1,000 IU/L, and a pericarditis was diagnosed when she came back to France. Pericarditis has been rarely reported after dengue fever, possibly because of a lack of detection in endemic areas. Some ten cases have been reported from Malaysia, Sri Lanka, Singapore, Brazil in total and one case in a French traveller returning from Guadeloupe [15-18].

The two cases here should remind us that dengue screening should be performed in malaria-negative travellers with history of fever returning from Africa [1,19]. For systematic screening of returning travellers for dengue fever, rapid diagnostic tests (as commonly done for malaria) are available and should be used. Rapid and early detection of cases could allow implementing measures to prevent further spread i.e. mosquito control around the residence of the returning travellers in areas where competent vectors are present and adapting the prevention message for travellers who wish to visit Africa. 

It is a well-known fact that travellers may serve as sentinels to local risks and this has been proven in numerous instances. In countries with scarce public health reporting, they may inform the international community on the onset of epidemics. Data from the African continent on dengue fever illustrates this phenomenon: dengue infections have been detected in 34 African countries, and for 12 of them the only available information was from travellers [2]. At the time we diagnosed our first case, the outbreak in Burkina Faso had not yet been notified by the WHO and the serotype involved was not known. 

An international festival of theatre named ’Les récréâtrales’ took place in Ouagadougou, from 29 October to 5 November. This festival occurs every two years in the capital between October and November, the months in which all previous dengue outbreaks were described. Our second patient mentioned that two other members of the festival staff were diagnosed with dengue fever during her stay. This festival takes place in a popular district of Ouagadougou (Bougsemtenga) and some presentations are organised in familial yards surrounding houses. A recent entomological survey in Ouagadougou identified that these yards were major places of vectors’ breeding sites [20]. The most frequent breeding sites identified were water storage containers, garbage such as food tins, and tyres [20]. In this survey, *Aedes aegypti* specimens were captured from breeding sites but no *Ae. albopictus* was identified [20]. *Aedes* mosquitoes bite during daytime. Hence, clinicians should remind travellers to endemic areas, including those in Africa, of the importance to protect themselves against mosquito bites during the day.
